# Supplementary material for: Associations between soil-transmitted helminth infections and physical activity, physical fitness, and cardiovascular disease risk in primary schoolchildren from Gqeberha, South Africa
Source: PLoS Negl Trop Dis. 2023 Oct 13;17(10):e0011664. doi: 10.1371/journal.pntd.0011664 (PMC10575529; doi:10.1371/journal.pntd.0011664)
Supplement: S1 Table — (DOCX) [file pntd.0011664.s001.docx]

**Table S1 (Supplement).** Differences between children with complete vs. incomplete data

|  | **Complete data (n=680)** | **Incomplete data (n=624)** | **Statistics** | | |
| --- | --- | --- | --- | --- | --- |
| **Participant characteristics** | **M (SD)** | **M (SD)** | **F** | **p** | **η2** |
| Age (years) | 8.20 (1.42) | 8.40 (1.47) | 5.53 | 0.019 | 0.005 |
| Height (cm) | 123.98 (9.07) | 125.32 (9.25) | 5.83 | 0.016 | 0.005 |
| Weight (kg) | 24.72 (6.13) | 26.23 (7.99) | 12.94 | < 0.001 | 0.011 |
| BMI (kg/m^2^) | 15.89 (2.35) | 16.42 (3.16) | 10.28 | 0.001 | 0.009 |
| Grip strength (kg) | 11.40 (4.87) | 11.11 (4.56) | 1.06 | 0.303 | 0.001 |
| VO_2_max (ml/kg/min) | 47.56 (3.48) | 47.41 (4.49) | 0.42 | 0.517 | 0.000 |
| MVPA (min/day) | 80.07 (26.37) | 85.64 (30.51) | 9.34 | 0.002 | 0.009 |
| Clustered CVD risk | -0.05 (2.68) | 0.20 (2.59) | 1.12 | 0.290 | 0.001 |

Note*.* BMI= Body mass index, VO_2_max = Cardiorespiratory fitness, MVPA= Moderate-to-vigorous physical activity, *η^2^* ***=*** Partial eta squared: 0.01 = small, 0.06 = medium, 0.14 = large effect size.
