# Supplementary material for: Associations between soil-transmitted helminth infections and physical activity, physical fitness, and cardiovascular disease risk in primary schoolchildren from Gqeberha, South Africa
Source: PLoS Negl Trop Dis. 2023 Oct 13;17(10):e0011664. doi: 10.1371/journal.pntd.0011664 (PMC10575529; doi:10.1371/journal.pntd.0011664)
Supplement: Attachment — Submitted filename: Point-by-point revision report.docx [file pntd.0011664.s002.docx]

Nqweniso et al.

Manuscript number: PLOS Neglected Tropical Diseases submission (PNTD-D-22-01288)

Associations between soil-transmitted helminth infections and physical activity, physical fitness, and cardiovascular disease risk in primary schoolchildren from Gqeberha, South Africa

**Point-by-point response**

Gqeberha, 21 July 2023

Dear Eva Clark

We refer to your e-mail dated 13 March 2023 and thank you and the three external referees very much indeed for the opportunity to submit a revision of the aforementioned manuscript for consideration for publication PLOS Neglected Tropical Disease. We found the comments of the reviewers very helpful and constructive in crafting a revision.

As per your request, we are submitting the document electronically. Below, we respond to each point raised by the reviewers. In the edited manuscript, we have highlighted all changes in yellow. If we can provide any additional information, or make any additional changes, please do not hesitate to let me know.

Yours sincerely,

Siphesihle Nqweniso (on behalf of all authors)

**Methods**

**Reviewer #1:** The objectives of the study are clear. Power calculations are for the original study and this analysis is just from the baseline data. No concerns with ethics or regulatory. Measurement of variables of interest seem appropriate. In the intro, the causal pathway of physical activity, CVD risk and STH infection is not clear, which would change which variables are exposures versus outcomes. For the purpose of this study, a hypothesis could be stated to help the reader understand why the analysis is being chosen as it is. Based on the analysis, the STH infection is being treated as an exposure. If infection is only being evaluated as a dichotomous variable, why not use a chi-square test rather than an ANOVA?

Response: We thank Reviewer #1 for the encouraging feedback regarding our research. Given the cross-sectional design of the study, it is not possible to infer any conclusions regarding cause and effect. Hence, in a theoretical model, both STH or the other variables (physical activity, fitness, CVD-risk) could be seen as exposure or outcome variable. In line with your recommendation, we have formulated three hypotheses at the end of the introduction section (see lines **128-134**). Furthermore, we have highlighted more clearly in the discussion section (among the limitations) that no causal relationships were tested. Theoretically, it is also possible that the relationship between these variables is bidirectional. We have also mentioned that longitudinal research is needed to examine the directionality of the relationship.

**Reviewer #2**: There are some questions regarding the method:

1. In comparison to their non-infected peers, children infected with STH had lower mean grip strength scores, but higher mean VO2max estimation and higher levels of MVPA.

Response: Thank you for the comment. Yes, infected children had lower mean grip strength scores, but higher mean VO_2_max estimation and higher MVPA. The higher VO_2_max might be supported by the lower BMI of the infected children as they completed more laps in the shuttle run test, which in turn, may be a disadvantage for strength as low weight is associated with lower grip strength scores.

2. Why did the authors choose this measurement? Would it be the lifestyle that cause this difference? Can the infected children have more outdoor activities than the non-infected as mentioned?

Response: We were not fully sure what the reviewer means with “this measurement”. The focus on STH infection was chosen because of previous studies in the same area that have shown a high prevalence in children from the one study area compared to the other areas. We agree with the reviewer’s assumption that the environment and cultural lifestyle of these children can cause the difference. However, due to the cross-sectional nature of our data, we need to be cautious with causal interpretation of our data. The findings of this paper are in line with previous studies from the same geographic areas (with older children), which have shown that a higher infection rate was reported in children with high self-reported physical activity, which might indicate that they participate in more outdoor activities (Gerber, Muller, Walter *et al*, 2018). In the end, our research helps to understand the risks and benefits of physical activity, i.e., what further preventive measures need to be taken, when promoting physical activity in these settings.

3. Is the lower grip strength score associated with energy intake or excessive energy uses?

Response: Reviewer #2 raises an important question, although this question goes beyond the scope of this paper. This is an interdisciplinary study and there are researchers focusing on dietary behaviour and they are still in the process of analysing this data. To avoid an overlap or interfering with their data, we decided to not include this variable in our analysis even though research has identified a correlation between grip strength and dietary behaviour. At a later stage, we will consider including dietary behaviour in our analyses to test whether grip strength correlates with dietary behavior or with total energy expenditure (accelerometry data) and whether infected vs. non-infected children differ in these variables.

4. Any information collected on their habits or lifestyle beside the MVPA?

Response: Yes. The study also collected subjective information on physical activity participation during school and after school hours. In this paper, we did not include this information in our data analysis since our focus is on “objectively” assessed physical activity (via accelerometry). We assessed self-reported physical activity with the parental reports (assessed with one item: *Physical activity can be done in sports, school activities, playing with friends or walking to school. Over the past 7 days, on how many days was your child physically active for a total of at least 60 minutes [1 hour] per day*?).

Self-reported physical activity has some limitations, such as inaccuracy due to recall bias, social desirability bias and question misunderstanding (Sallis, 1991). A study comparing self-reported with objectively measured physical activity data in schoolchildren from marginalised communities in Gqeberha reported a positive but weak correlation between these two methods (Arnaiz, Guntlisbergen, Infanger, Gerber *et al*, in-print). These findings are in agreement with previous studies that also reported discrepancies between self-reported and objective methodologies in estimating physical activity in children. We are also aware that parents might have not reported accurate information about their child’s physical activity participation. The use of ecological monetary assessment (EMA) surveys has been reported as a more superior method of assessing children’s physical activity levels compared to self-reported surveys, due to its strong association with objectively measured physical activity (Brito, Loth, Tate & Berge, 2020). Future research can consider using EMA instead of self-reported data.

5. The authors have discussed this later in the discussion part, however if they had the data on characteristics of the physical activities of the school and the non-infected children, that might be useful information. If those children have similar physical activities levels, then it might be interesting to understand why they showed different results than other study.

Response: We decided not to include these data for two reasons. First, our focus was on device-based physical activity. Second, the question of validation of our self-report data has not been conclusively answered at the time of submission and resubmission (see answer 4 above). Thus, for the time being, the stigma remains that potential biases could not be excluded in these data.

6. More than 80% of the infected children has coinfection. Any plan on testing the stools with a more sensitive measurement such as PCR?

Response: Thank you for the question. We are aware that the Kato-Katz diagnostic method has limitations in its ability to pick up low-intensity infection, which can be the reason for the low number of light-intensity infections detected in the current study. A more sensitive measurement would be ideal and can be considered in future studies. We have mentioned this as a limitation and recommendation for future research in the discussion section (see lines **497-499**).

7, The VOmax and the MVPA results seem to show different between schools. Is there any characteristics information collected on the schools? The authors indeed did an analyses in one school with relatively high helminth prevalence. But I wonder whether the school itself and the children went to this school also had different characteristic than other schools.

Response: Thank you for this question. As shown in Table 1, children from different schools did differ with regard to sex, age and BMI, with no differences in socio-economic status (SES). Given these significant between-school differences in these variables, we decided to control for these potential confounders in the models. Tables 2a, 2b and 3 now show results for the unadjusted and adjusted models. STH infection in South Africa is highly dependent on the area children live in.

--------------------

**Results**

**Reviewer #1**: With almost half the cohort being dropped for missing data, some description of that group would be helpful to understand how missing data may be biasing the results. Table 1 by school is confusing as there is almost no STH in the schools except one. Is there a more informative way to structure this table? Many of the outcomes vary by age. How do the outcomes vary by gender. Is it shown how age and BMI are associated with STH infection? Table 1 by STH infection (yes/no) excluding your outcome variables would help indicate why and which variables you are treating as confounders.

Response: Thank you. We have now tested whether differences existed between children with complete vs. incomplete data with regard to age, height, weight, BMI, grip strength, VO_2_max, MVPA and clustered CVD risk with ANOVAs. We present the outcomes of these analyses as supplementary online material. As shown, compared to children with complete data, children with incomplete data were significantly older, had higher scores for height, weight, BMI and MVPA but the effect size was small (η²≤0.01). We found no significant group differences regarding grip strength and VO_2_max (see lines **280-287** and Table S1).

For table 2, adjusted and unadjusted outcomes in one table would be helpful for comparison. And what is adjusted for, would relate to what is seen in table 1 as being associated with STH infection. Because STH infection numbers are so small, differences by intensity are even smaller. Did you consider combining light and moderate?

Response: Thank you for this suggestion. In line with your recommendation, we decided to provide F-, p- and η2-statistics for both the unadjusted and adjusted models. Adjusted models were controlled for sex, age and BMI. We have explained this in Tables 2a, 2b and 3. We understand that combing light and moderate infection intensity would increase the numbers and help statistically, but research has shown that light and moderate infection intensities differ in terms of how they affect their host. Disease burden cannot be identified from just being infected vs. non-infected. In our study, children infected with STH had higher cardiorespiratory fitness and physical activity levels, which might give misleading conclusions that being infected has beneficial effects. Hence, we decided to investigate the effects of the different infection intensities on the outcome variables.

--------------------

**Conclusions**

**Reviewer #1**: Drawing conclusions from intensity of infection and outcomes is challenged by small numbers. Focusing on conclusions based on infected or not may be more informative. In the paragraph starting on line 398, the causal pathway of physical activity, CVD and STH infection is hard to follow. The first sentence of the limitations states that the direction of the causality cannot be inferred due to the cross sectional study design. I agree but the analysis is based on an assumption so interpreting the results aligned with the hypothesis of the study may help with clarity.

Response: We agree that the small numbers provide a challenge when it comes to drawing conclusions. Given that infection intensity is important to consider in terms of disease burden, as well as the paucity in studies investigating how STH species and infection intensity affect physical activity, fitness and CVD risk, we decided to investigate these effects. Tahapary and colleagues found that the burden of infections, in terms of the number of helminth species as well as the infection intensity, can influence the effect of anthelmintic treatment on insulin resistance (Tahapary, de Ruiter, Martin *et al*, 2017).

**Reviewer #2**: The authors have addressed the conclusion, the limitation and the strengths of the study, however they might want to explore studies related to the topic they raised such as studies done in Indonesia.

Response: Thank you. We have performed again a thorough review of the literature and have summarised the results of these studies in the introduction (see lines **106-114**) and discussion section (see lines **444-447**).

**Summary and General Comments**

**Reviewer #1**: The study is interesting and the authors presented the information nicely. Some refinement as noted above would help with clarity.

Response: Thank you for your positive feedback. We have addressed all points raised by the reviewers and hope that we were able to resolve all issues to their satisfaction.

**Reviewer #2**: Dear Authors,

Thank you for the opportunity to review this interesting manuscript. The manuscript can potentially be published and might contribute to limited information in this field.

1. In the abstract the authors mentioned: STH infections have been associated with negative consequences for child physical and cognitive development and wellbeing. Thus, the aim of this study was to determine the association of STH infections in schoolchildren from Gqeberha, focusing on physical activity, physical fitness, and clustered cardiovascular disease (CVD) risk score.

-> Why it is important to check CVD risk score in the study?

The explanation of the connection has been mentioned in the background text. One short connecting sentence might need to be added in the abstract to make it a bit more clear.

Response: Thank you. A sentence has been added in the abstract showing the connection between STH and CVD risk (see lines **24-26**).

2. Regarding the background and the discussion, I am surprised that th e authors did not come through studies by Pasha et al, Wiria et al and Tahapary et al, as they specifically concern on metabolic syndrome, cardiovascular risk (and atherosclerosis), and insulin sensitivity (resistance) in association with helminth infection and population at risk of epidemiological transition. They also did cohort study on this topic. They also have data on children and adult if that regarding some cardiovascular risk factors.

Response: Thank you. We have performed again a thorough review of the literature and have summarised the results of these studies in the introduction (see lines **106-114**) and discussion section (see lines **444-447**).

References

Arnaiz P, Guntlisbergen F, Infanger D, Gerber M, Adams L, Dolley D, et al. Association of accelerometry-based and self-reported physical activity with cardiovascular risk in South African children. S Afr J Sci. in-print.

de Brito JN, Loth KA, Tate A and Berge J. M. Associations Between Parent Self-Reported and Accelerometer-Measured Physical Activity and Sedentary Time in Children: Ecological Momentary Assessment Study. JMIR Mhealth Uhealth. 2020; 8(5):e15458. https://doi.org/10.2196/15458

Gerber M, Müller I, Walter C, du Randt R, Adams L, Gall S, et al. Physical activity and dual disease burden among South African primary schoolchildren from disadvantaged neighbourhoods. Prev Med. 2018; 112:104-110. https://doi.org/10.1016/j.ypmed.2018.04.001

Sallis JF. Self-report measures of children's physical activity. J Sch Health. 1991; 61(5):215-9. https://doi.org/10.1111/j.1746-1561.1991.tb06017.x

Tahapary DL, de Ruiter K, Martin I, Brienen EAT, van Lieshout L, Cobbaert CM, et al. Effect of Anthelmintic Treatment on Insulin Resistance: A Cluster-Randomized, Placebo-Controlled Trial in Indonesia. *Clin Infect Dis*. 2017;65(5):764-71. https://doi.org/10.1093/cid/cix416
